# Construction and clinical validation of a Team STEPPS-based discharge planning program for patients with inflammatory bowel disease

**DOI:** 10.3389/fmed.2026.1799752

**Published:** 2026-04-20

**Authors:** Pinju Lv, Min Li, Lisi Lao, Yaqin Li, Ruo Wang, Ping Zhang, Luzhou Xu

**Affiliations:** 1 Department of Nursing, Jiangsu Province Hospital of Chinese Medicine, Nanjing, Jiangsu, China; 2 Department of Gastroenterology, Jiangsu Province Hospital of Chinese Medicine, Nanjing, Jiangsu, China

**Keywords:** Delphi method, discharge planning program, inflammatory bowel disease, randomized controlled trial, Team STEPPS

## Abstract

**Background:**

The transition from hospital to home is a high-risk period for patients with Inflammatory Bowel Disease (IBD). This study aimed to develop and preliminarily validate a structured discharge planning program for patients with IBD by applying the Team STEPPS framework.

**Methods:**

This study employed a sequential two-phase, mixed-methods design. First, a Team STEPPS-based discharge planning program was rigorously developed using Delphi method between August and October 2023. Subsequently, a randomized controlled trial was conducted from October 2023 to March 2024 to validate the program. Ninety eligible inpatients were randomly assigned to an intervention group (*n* = 45) receiving the structured Team STEPPS program or a control group (*n* = 45) receiving standard care. The primary outcome was discharge readiness, assessed at the time of discharge. Secondary outcomes included the quality of discharge teaching, measured at discharge, and health-related quality of life, evaluated at baseline, discharge, and 30-day follow-up. Longitudinal outcomes analyzed via linear mixed-effects models.

**Results:**

All 90 enrolled participants completed the study. The Delphi process successfully achieved expert consensus, resulting in a structured discharge program with all items meeting predefined criteria (mean importance ≥ 4.0, coefficient of variation ≤ 0.25). In the subsequent randomized controlled trial, baseline characteristics were well-balanced. At discharge, the intervention group exhibited statistically superior outcomes, including significantly higher discharge readiness (103.0 ± 7.3 vs. 94.9 ± 4.7, *P* < 0.001) and quality of discharge teaching scores (183.6 ± 6.4 vs. 172.7 ± 5.0, *P* < 0.001). Longitudinal analysis of health-related quality of life revealed a significant group-by-time interaction effect (*P* < 0.001), with the intervention group demonstrating a greater magnitude of improvement. The model estimated an incremental benefit of the intervention of 9.2 points (95% CI: 5.7–12.7) at the 30-day follow-up compared to standard care.

**Conclusion:**

A Team STEPPS-based discharge planning program was successfully developed and shown to be effective, significantly improving discharge readiness, the quality of discharge teaching, and health-related quality of life in patients with IBD.

**Clinical trial registration:**

https://www.chictr.org.cn/index.html, identifier ChiCTR2501113716.

## 1 Introduction

Inflammatory bowel disease (IBD) is a chronic, relapsing-remitting, and incurable inflammatory disorder of the gastrointestinal tract (1). Its global prevalence continues to rise, contributing to a substantial and growing healthcare burden worldwide (2). The management of IBD is inherently complex and lifelong, extending beyond the control of mucosal inflammation (3). It necessitates continuous medical supervision, strict adherence to often complex medication regimens, significant lifestyle adjustments, and vigilant self-monitoring for signs of disease flare or complications. The unpredictable disease course, characterized by alternating periods of remission and active flares, frequently leads to hospital admissions for acute management, exacerbations, or surgical interventions (4). Consequently, the transition from hospital to home represents a critical and high-risk phase within the continuum of care. This discharge period is often marked by gaps in care coordination, medication errors, inadequate symptom recognition, and ultimately, preventable readmissions. Studies report that within 30 days of discharge, patients with IBD experience readmission rates significantly higher than those with many other chronic conditions, underscoring a systemic deficiency in current transitional care practices (5). Therefore, optimizing the discharge process is not merely a logistical concern but a fundamental clinical imperative to improve long-term outcomes, enhance quality of life, and reduce the economic burden of this chronic illness.

In many healthcare settings, traditional discharge planning, while well-intentioned, often fails to address the multifaceted needs of patients with IBD (6). Conventional approaches are frequently fragmented, provider-centric, and compressed into the final phase of hospitalization, typically consisting of standardized written instructions and a cursory medication review. This model lacks the depth, personalization, and continuity required for a dynamic chronic condition, thereby failing to adequately prepare patients for the challenges of autonomous self-management at home (7). Key deficiencies include insufficient education on recognizing early warning signs of flare-ups and managing complex therapies, poor coordination of care among multiple specialists leading to discontinuous follow-up, the systematic overlooking of psychosocial needs and post-discharge vulnerability, and a fundamental mischaracterization of discharge as a singular event rather than a preparatory process. This gap between institutional care and home management directly contributes to adverse outcomes, including non-adherence to treatment, delayed response to complications, increased emergency department visits, and early hospital readmission (8). Consequently, there is a clear and pressing need for a structured, evidence-based, and patient-centered discharge planning program specifically tailored to the unique clinical trajectory of the patient with IBD.

To address these critical deficiencies in current discharge practices for IBD, a structured framework that enhances team coordination and patient engagement is essential. The Team Strategies and Tools to Enhance Performance and Patient Safety (Team STEPPS) framework, developed by the Agency for Healthcare Research and Quality (AHRQ) and the U.S. Department of Defense, provides a validated foundation (9). Originally designed to improve safety and outcomes in high-stakes environments by strengthening teamwork, its core principles, which include Leadership, Situation Monitoring, Mutual Support, and Communication, are directly applicable to the complex coordination required during care transitions (9). Integrating Team STEPPS into discharge planning shifts the paradigm from a siloed, task-oriented process to a dynamic, team-based model where patients and families are central members. In the context of IBD, this framework can standardize how multidisciplinary teams collaboratively develop and execute unified care plans (10). Tools such as SBAR (Situation-Background-Assessment-Recommendation) can structure handoff communication, while Briefs, Huddles, Check-Backs, and Call-Outs can align team goals and enhance medication safety and patient education (11, 12). Although Team STEPPS has demonstrated efficacy in settings such as surgery and emergency care, its systematic application to design and evaluate a structured discharge program for a chronic medical population like IBD remains an underexplored area (13). Current initiatives may incorporate teamwork elements but often lack rigorous methodology in both program development and evaluation.

Therefore, this study employs a two-phase, mixed-methods approach aimed at developing and preliminarily validating a Team STEPPS-based discharge planning program for patients with IBD. The first phase utilizes the Delphi method to integrate evidence and multidisciplinary expert consensus for the systematic construction of the program’s core components. The second phase involves a controlled clinical pilot to assess the program’s feasibility and preliminary effectiveness in a real-world clinical setting. The ultimate goal is to translate the theoretical strengths of teamwork and safety culture into a concrete, protocolized intervention. This aims to enhance the quality of transitional care for this vulnerable patient population and to reduce preventable morbidity and healthcare utilization following hospital discharge.

## 2 Materials and methods

### 2.1 Study design

This study employed a two-phase, mixed-methods design. The first phase utilized the Delphi method to develop a Team STEPPS-based discharge planning program for patients with IBD. The second phase was a randomized controlled trial. Eligible patients were randomly assigned to either a control group receiving standard care or an intervention group implementing the Team STEPPS-based discharge planning program. This randomized controlled trial aimed to validate the feasibility and effectiveness of the Team STEPPS-based discharge planning program.

### 2.2 Delphi study

The Team STEPPS-based discharge planning program for patients with IBD was developed using a modified Delphi method (14). First, a multidisciplinary research team was formed, comprising gastroenterologists, IBD specialist nurses, and clinical research methodologies. This team conducted a systematic review of the existing literature, with a focus on identifying current challenges in the transitional care of discharged IBD patients, the core components of the Team STEPPS framework, and its potential application in chronic disease management. Based on this evidence synthesis and subsequent team discussions, a preliminary version of the Team STEPPS-based discharge planning program was drafted, which served as the foundation for the first-round Delphi questionnaire.

A panel of 16 experts was selected from six key fields: gastroenterology, nursing, psychology, clinical nutrition, traditional Chinese medicine, and chronic disease management. The inclusion criteria for experts were: (1) holding an associate senior professional title or higher; (2) possessing no less than 10 years of clinical, nursing, or research experience in IBD; and (3) being familiar with patient education, chronic disease management, or interdisciplinary teamwork.

The expert consultation was conducted anonymously via questionnaire over two rounds between August and October 2023. The questionnaires contained both open-ended and closed-ended questions. Experts were asked to rate the importance of each program component on a 5-point Likert scale (1 = not important at all, 5 = extremely important) and provide written suggestions for modification. The importance scores for each item were presented as mean ± standard deviation (SD) to reflect the central tendency of expert opinions. The response rate for each round was calculated to assess panel engagement. The authority coefficient (Cr) of the experts was determined by calculating the average of their self-assessed judgment coefficient (Ca) and familiarity coefficient (Cs), using the formula: Cr = (Ca + Cs)/2 (15). After the first round, the research team calculated the mean score, SD, and coefficient of variation (CV) for each item and synthesized all qualitative feedback. Items with significant disagreement were revised or consolidated, leading to the development of the second-round questionnaire. In the second round, experts received a summary of the first-round statistical analysis and the rationale for revisions before re-rating the adjusted items. A pre-defined consensus criterion was applied: an item was considered to have reached consensus if its mean importance score was ≥ 4.0 and its CV was ≤ 0.25 (15). Furthermore, Kendall’s *W* was calculated to assess the degree of agreement among the expert panel on the importance ratings of all items in the second round.

The research team analyzed the second-round data to identify all items meeting the consensus threshold. For items that did not fully meet the pre-set criteria but were deemed critical by the core research team, a final decision was made by integrating the expert written comments. All consensus-based items were systematically integrated to form the final, structured, and operational “Team STEPPS-based Discharge Planning Program for IBD Patients” (Table 1), which was subsequently implemented in the randomized controlled trial.

**TABLE 1:** Team STEPPS-based discharge planning program for IBD patients.

Sections	Nursing measures	Responsible person	Tools/methods	Importance scores	CV
Admission assessment	1. Designate a team leader and clearly define the roles and responsibilities of team members.	Team leader	Team role assignment chart	4.38 ± 0.53	0.121
	2. Use the SBAR tool to document the patient’s condition upon admission and share it with team members.	Doctors, nurses	SBAR template, electronic health record system	4.19 ± 0.48	0.115
	3. Assess the patient’s disease activity, nutritional status, psychological state, and family support situation.	All team members	CDAI score, mayo score, nutrition screening tool	4.81 ± 0.58	0.21
	4. Team members jointly discuss the assessment results and develop a preliminary discharge plan.	All team members	Multidisciplinary team meeting	4.44 ± 0.61	0.137
Plan development	1. The team leader organizes multidisciplinary meetings to develop personalized discharge plans.	Team leader	Multidisciplinary team meeting	4.69 ± 0.55	0.117
	2. Use the SBAR tool to clarify the patient’s treatment goals, discharge date, and follow-up plan.	Doctors, nurses	SBAR Template, electronic health record system	4.31 ± 0.50	0.116
	3. Monitor changes in the patient’s condition and adjust the discharge plan as needed.	All team members	Daily medical record form	4.88 ± 0.59	0.121
	4. Nutritionists collaborate with physicians to develop dietary plans, while nurses provide psychological support.	Nutritionist, nurses	Personalized meal plans, psychological support programs	4.56 ± 0.66	0.145
Teamwork	1. The team leader monitors task completion to ensure each step is executed according to plan.	Team leader	Task list, team collaboration scorecard	4.50 ± 0.62	0.138
	2. Hold regular team meetings to update patient conditions and task progress using the SBAR tool.	All team members	Regular meetings, SBAR template	4.25 ± 0.47	0.111
	3. Continuously monitor the patient’s condition, medication response, and psychological state to promptly identify and address any issues.	All team members	Daily condition record form, medication reaction monitoring form	4.94 ± 0.60	0.121
	4. Team members support each other and work together to resolve issues related to patient discharge preparations.	All team members	Internal team support mechanism	4.63 ± 0.68	0.147
Patient education	1. The team leader coordinates patient education content to ensure information is consistent and comprehensive.	Team leader	Patient education handbook	4.44 ± 0.56	0.126
	2. Explain the discharge plan to patients and their families using plain language to ensure they understand and cooperate.	Nurses	One-on-one tutoring, educational handbook	4.88 ± 0.57	0.117
	3. Assess the patient and family’s knowledge retention and supplement educational content as needed.	Nurses	Knowledge assessment questionnaire	4.69 ± 0.71	0.151
	4. Provide psychological support and information on social resources to patients and their families to help them cope with the stress of illness.	Nurses, volunteer	Psychological support programs, social resource connections	4.81 ± 0.64	0.133
Post-discharge follow-up	1. The team leader develops a follow-up plan, specifying the frequency and content of follow-ups.	Team leader	Follow-up schedule	4.38 ± 0.54	0.123
	2. Maintain communication with patients through telephone follow-ups, cloud clinics, and other methods to monitor their health status.	Doctors, nurses	Telephone follow-up form, cloud clinic	4.13 ± 0.45	0.109
	3. Continuously monitor patients’ disease activity, medication adherence, and quality of life, and adjust management strategies as needed.	All team members	Risk assessment form, quality of life score sheet	4.75 ± 0.58	0.122
	4. Provide emergency support to patients to ensure they can promptly receive assistance if issues arise after discharge.	All team members	24-h hotline and online consultation platform	4.56 ± 0.67	0.147
Emergency support	1. The team leader develops an emergency response plan, clearly defining each team member’s responsibilities and action steps during emergencies.	Team leader	Emergency response manual	4.69 ± 0.60	0.128
	2. Through a 24-h hotline and online consultation platform, ensure patients can promptly reach the medical team.	All team members	24-h hotline and online consultation platform	4.31 ± 0.49	0.114
	3. Monitor patients for emergencies, provide timely support, and document the outcomes of interventions.	All team members	Emergency record form	4.88 ± 0.61	0.125
	4. Team members collaborate to jointly address patient emergencies.	All team members	Team collaboration scorecard	4.50 ± 0.65	0.144

In the final analysis, all items in the program achieved a mean importance score of ≥ 4.0 and a CV of ≤ 0.25 (Table 1). The Kendall’s *W* for the second-round ratings was calculated to be 0.925 (*P* < 0.001), indicating a strong and significant consensus among the expert panel. The response rate was 100% for both questionnaire rounds, indicating a high level of panel participation. In the second round, the experts’ Ca was 0.923, their Cs was 0.905, resulting in an Cr of 0.914, which signifies a high degree of expert authority for the panel.

### 2.3 Participants

The required sample size was determined through an a priori power analysis for the primary outcome of discharge readiness, employing a two-independent-samples design. Parameters were set with a two-sided alpha (α) of 0.05 and 90% statistical power (β = 0.10). An effect size (δ/σ) of 0.8 for the expected group difference was adopted from prior research (16). The following formula was applied to calculate the minimum sample size per group (N) (17):


$$\text{N}=2\,{\left[\left({\text{t}}_{\frac{\alpha }{2}}+{\text{t}}_{\frac{\beta }{2}}\right)\,\sigma /\delta \right]}^{2}$$

This calculation yielded a requirement of 34 participants per group. To account for an estimated 20% attrition rate, the sample size was inflated, resulting in a final planned enrollment of 43 participants per group, for a total of 86 participants.

This study was conducted at Jiangsu Provincial Hospital of Traditional Chinese Medicine between October 2023 and March 2024. A total of 90 eligible inpatients with IBD were enrolled as study participants. Upon admission and after confirming eligibility, patients were allocated at a 1:1 ratio to either the intervention group or the control group using a random number table, with 45 patients in each group.

Inclusion criteria: (1) age ≥ 18 years; (2) meeting the clinical diagnostic criteria for Crohn’s disease or ulcerative colitis (18); (3) being conscious and possessing basic cognitive and communication abilities to comprehend the study content; (4) availability for a follow-up period of at least 30 days post-discharge; (5) voluntary participation and provision of written informed consent. Exclusion criteria: (1) comorbid severe extraintestinal complications, malignancy, psychiatric disorders, or cognitive impairment; (2) pregnancy or lactation; (3) participation in other similar interventional studies or discharge preparation-related surveys within the past 3 months; (4) plans to relocate away from the local area after discharge, which would preclude completion of follow-up.

**FIGURE 1:**
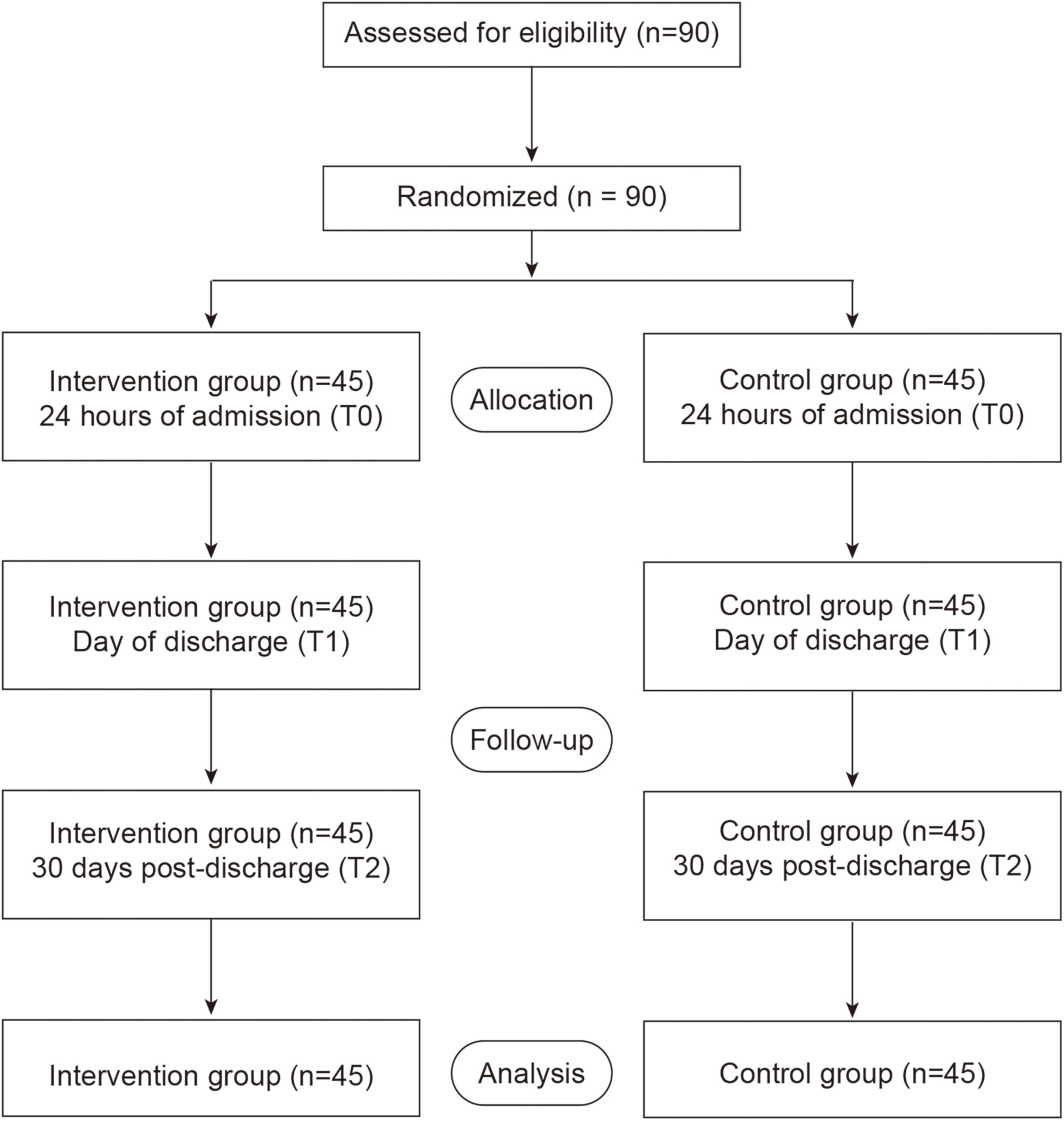
Study design flowchart.

All analyses were performed on an intention-to-treat basis, meaning that all randomized participants were included in the final analysis and were analyzed according to their original group assignment. Because there were no dropouts or withdrawals during the study, the intention-to-treat population was identical to the per-protocol population. The study design flowchart is shown in Figure 1.

The study protocol was approved by the Ethics Committee of Ethics Committee of Jiangsu Province Hospital of Chinese Medicine (Approval No. YJZ202652) and the trial was registered in the Chinese Clinical Trial Registry (Registration No. ChiCTR2501113716). All participants received comprehensive information regarding the study’s purpose, procedures, potential risks, and rights prior to enrollment and provided written informed consent. The entire research process strictly adhered to the ethical principles of the Declaration of Helsinki.

### 2.4 Intervention

The control group received the standard inpatient care protocol. This included foundational nursing services to ensure a safe and comfortable hospitalization environment, specialized nursing guidance tailored to the individuals condition (encompassing medication management, dietary advice, and psychological support), and transitional care support. To facilitate continuous communication and enhance post-discharge follow-up, an online platform was established using the WeChat application.

In contrast, the intervention group received the standard care plus a comprehensive, structured discharge planning program rigorously developed based on the Team STEPPS framework. This program was implemented by a designated multidisciplinary team, including a team leader, gastroenterologists, nurses, a clinical pharmacist, and a dietitian. Prior to the study implementation, all members of the multidisciplinary team underwent a standardized 4-h training session on the Team STEPPS framework and the specific study protocol. The training, led by the principal investigator, included didactic lectures, video demonstrations of Team STEPPS tools, and role-playing scenarios simulating IBD discharge planning. A training manual and quick-reference pocket cards were provided to all team members. The intervention unfolded across six sequential phases: Admission Assessment, Plan Formulation, Team Collaboration, Patient Education, Post-Discharge Follow-up, and Emergency Support. Structured Team STEPPS tools were employed throughout. During Admission Assessment, the team leader clarified roles using a responsibility matrix, while clinicians used SBAR templates to document and share initial patient information. Disease activity, nutritional status, and psychosocial support were assessed collaboratively to draft a preliminary discharge plan. In the Plan Formulation phase, the leader convened multidisciplinary meetings to finalize a personalized plan, with SBAR used to define treatment goals and timelines. Daily patient monitoring informed ongoing plan adjustments. The Team Collaboration phase was characterized by the leader overseeing task completion via checklists, regular team huddles using SBAR for updates, and mutual support mechanisms for problem-solving. Patient Education was coordinated by the leader to ensure consistency, with nurses providing one-on-one instruction using a standardized, plain-language education booklet developed specifically for this study, which covered topics such as medication management, dietary guidance, flare recognition, and when to seek help. Nurses assessed patient comprehension using the “teach-back” method. The Post-Discharge Follow-up and Emergency Support phases involved structured tele-follow-ups, continuous remote monitoring, and guaranteed access to the team via a 24-h hotline, with predefined emergency protocols.

**FIGURE 2:**
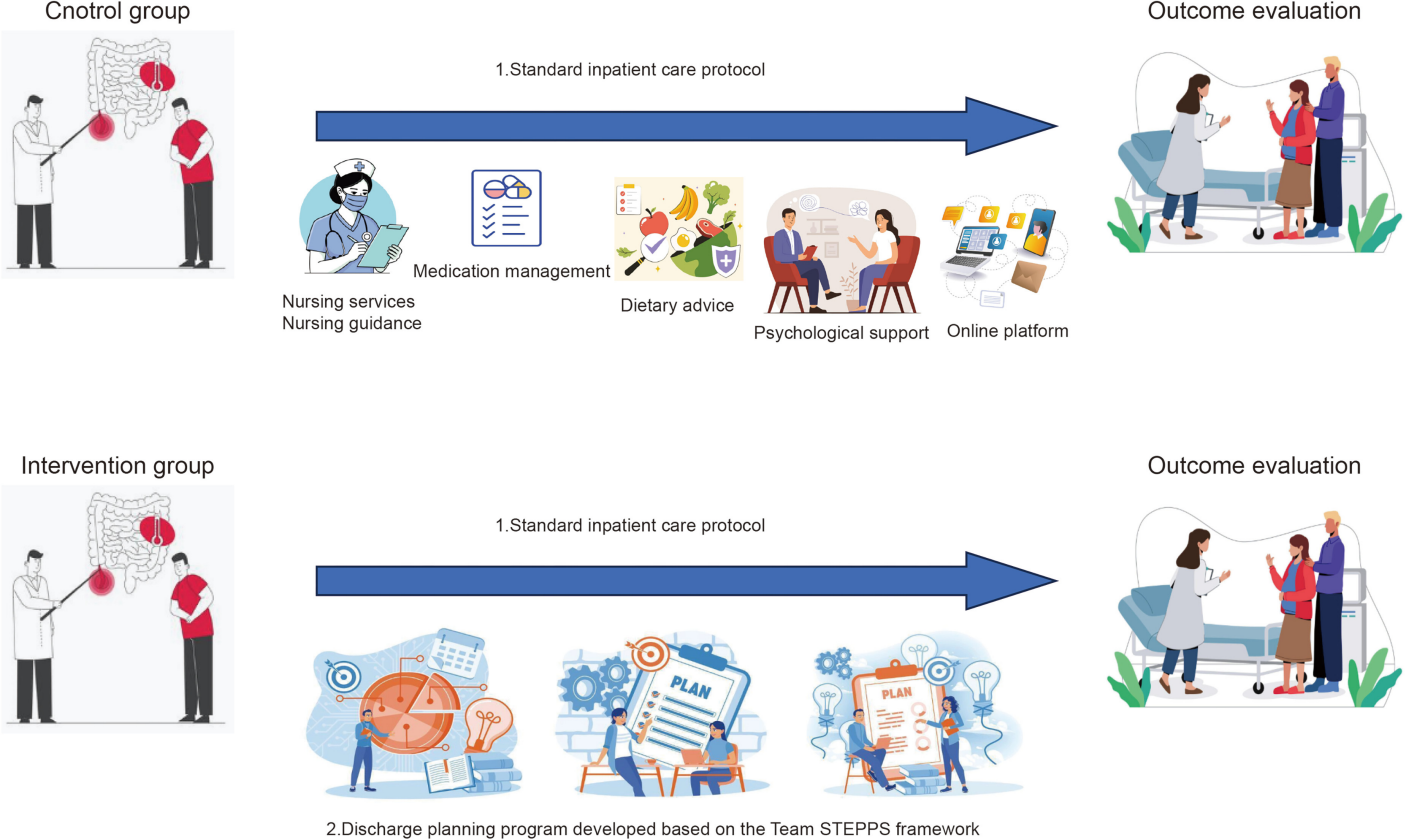
Flowchart of the care pathways for the control and intervention groups.

To ensure intervention fidelity, multiple strategies were employed. (1) All intervention components and materials were manualized. (2) As described above, all team members received standardized training. (3) A designated research coordinator independently reviewed a random sample of 20% of patient records and audio-recorded team huddles (with consent) against a fidelity checklist to ensure core components were delivered as planned. Feedback was provided to the team monthly. (4) Patient comprehension was verified using the teach-back method during education sessions. A schematic diagram summarizing and contrasting the care pathways for both the control and intervention groups is provided in Figure 2.

### 2.5 Data collection

Data collection was conducted at three predefined time points by a trained research assistant blinded to group allocation. Baseline data (T0), obtained within 24 h of admission, encompassed demographic and clinical characteristics extracted from medical records and patient interviews. These variables included age, gender, family annual income, marital status, educational level, disease duration, disease type (Crohn’s disease or ulcerative colitis), and reason for admission. The reason for admission was retrieved from electronic medical records and classified into three categories: Flare (acute exacerbation requiring intensified medical therapy); Surgical complication (conditions necessitating surgical evaluation/intervention, e.g., obstruction, abscess, fistula); and Other (e.g., infection, thromboembolism, medication adverse effects, nutritional support, or non-IBD causes). The primary outcome was discharge readiness. Secondary outcomes included the quality of discharge teaching and health-related quality of life.

Discharge readiness, was evaluated on the day of discharge (T1) using the validated Chinese version of the Readiness for Hospital Discharge Scale (RHDS) (19). This 12-item instrument assesses three dimensions: personal status, coping ability, and anticipated support. Each item is rated on a 0–10 Likert scale. The scale has demonstrated good internal consistency reliability in validation studies, with a reported Cronbach’s α of 0.89. The quality of discharge teaching was measured on T1 using the Chinese version of the Quality of Discharge Teaching Scale (QDTS) (20). This 24-item tool comprises three dimensions: needed content, received content, and delivery method/effectiveness, all scored on a 0–10 scale. The discrepancy between scores from the first two dimensions reflects the degree of met needs, while the total score indicates overall teaching quality, with higher scores denoting better quality. The scale has shown excellent psychometric properties, with a Cronbach’s α of 0.924.

Health-related quality of life was assessed at T0, T1, and T2 (30 days post-discharge) using the 32-item Inflammatory Bowel Disease Questionnaire (IBDQ) (21). The instrument contains 32 items across four domains: systemic symptoms, bowel symptoms, social function, and emotional function. Each item is rated on a 1–4 scale, yielding a total score ranging from 32 to 128, where higher scores correspond to better quality of life. Validation studies in Chinese populations have confirmed the scale’s suitability, reporting a total Cronbach’s α of 0.949 and subscale coefficients all exceeding 0.7.

### 2.6 Statistical analysis

Statistical analyses were performed using SPSS 26.0. Categorical variables are presented as frequencies and percentages (*n*, %) and were compared using the chi-square. Continuous variables were tested for normality with the Shapiro-Wilk test. Normally distributed data are expressed as mean ± standard deviation (SD) and compared using the independent samples *t*-test. To analyze the longitudinal outcomes across the three assessment time points (T0, T1, T2), a linear mixed-effects model (LMM) was employed as the primary analytical framework (22). This model was chosen for its flexibility in handling repeated measures data and ability to account for within-subject correlation. The fixed effects in the model included time (as a categorical factor), group (intervention/control), and the time-by-group interaction. For all analyses, a two-sided *P* < 0.05 was considered statistically significant.

## 3 Results

### 3.1 Baseline characteristics

Table 2 summarizes the baseline characteristics of all participants. No statistically significant differences were observed between the intervention and control groups for any of the measured variables, including age, gender, family annual income, marital status, educational level, disease duration, disease type, and reason for admission (all *P* > 0.05), confirming successful randomization and baseline comparability.

**TABLE 2:** Characteristics of patients [n (%)].

Variables	Intervention group (*n* = 45)	Control group (*n* = 45)	χ ^2^	*P*
Age			0.054	0.973
18–45	18 (40.0)	17 (37.8)		
46–60	19 (42.2)	20 (44.4)		
>60	8 (17.8)	8 (17.8)		
Gender			0.179	0.673
Female	22 (48.9)	20 (44.4)		
Male	23 (51.1)	25 (55.6)		
Family annual income			0.421	0.810
<30,000 CNY	16 (35.6)	16 (35.6)		
≥30,000 CNY	22 (48.9)	24 (53.3)		
Uncertain	7 (15.6)	5 (11.1)		
Marital status			0.223	0.637
Married	32 (71.1)	30 (66.7)		
Unmarried /other	13 (28.9)	15 (33.3)		
Educational level			1.287	0.257
≤High school	24 (53.3)	19 (42.2)		
>High school	21 (46.7)	26 (57.8)		
Disease duration			0.416	0.812
≤1	20 (44.4)	18 (40.0)		
2–5	19 (42.2)	22 (48.9)		
> 5	6 (13.3)	5 (11.1)		
Disease type			0.403	0.525
Ulcerative colitis	23 (51.1)	26 (57.8)		
Crohn’s disease	22 (48.9)	19 (42.2)		
Reason for admission			0.251	0.882
Flare	30 (66.7)	28 (62.2)		
Surgical complication	10 (22.2)	12 (26.7)		
Other	5 (11.1)	5 (11.1)		

### 3.2 Discharge readiness and quality of discharge teaching of patients

At discharge (T1), for discharge readiness, the total score was significantly greater in the intervention group (103.0 ± 7.3) compared to the control group (94.9 ± 4.7) (*P* < 0.001). Personal status, anticipated support, and coping ability also showed statistically significant improvements (all *P* < 0.001).

Similarly, the total score for the quality of discharge teaching was significantly higher in the intervention group (183.6 ± 6.4) compared to the control group (172.7 ± 5.0) (*P* < 0.001). Significant between-group differences were also found in the received content and delivery method/effectiveness subscales (all *P* < 0.001). The difference in the needed content subscale was smaller but remained statistically significant (*P* = 0.023). Detailed results are presented in Table 3.

**TABLE 3:** Discharge readiness and quality of discharge teaching of patients (mean ± SD).

Variables	Intervention group (*n* = 45)	Control group (*n* = 45)	*T*	*P*
Discharge readiness	103.0 ± 7.3	94.9 ± 4.7	-6.357	< 0.001
Personal status	15.6 ± 3.0	12.8 ± 2.1	-5.000	< 0.001
Anticipated support	18.2 ± 3.6	15.4 ± 2.4	-4.257	< 0.001
Coping ability	69.3 ± 3.5	66.6 ± 3.5	-3.685	< 0.001
Quality of discharge teaching	183.6 ± 6.4	172.7 ± 5.0	-9.099	< 0.001
Needed content	49.6 ± 3.4	48.0 ± 3.2	-2.310	0.023
Received content	43.6 ± 3.4	39.6 ± 3.2	-3.978	< 0.001
Delivery method/effectiveness	90.5 ± 2.7	85.1 ± 1.9	-10.926	< 0.001

### 3.3 Health-related quality of life of patients

**FIGURE 3:**
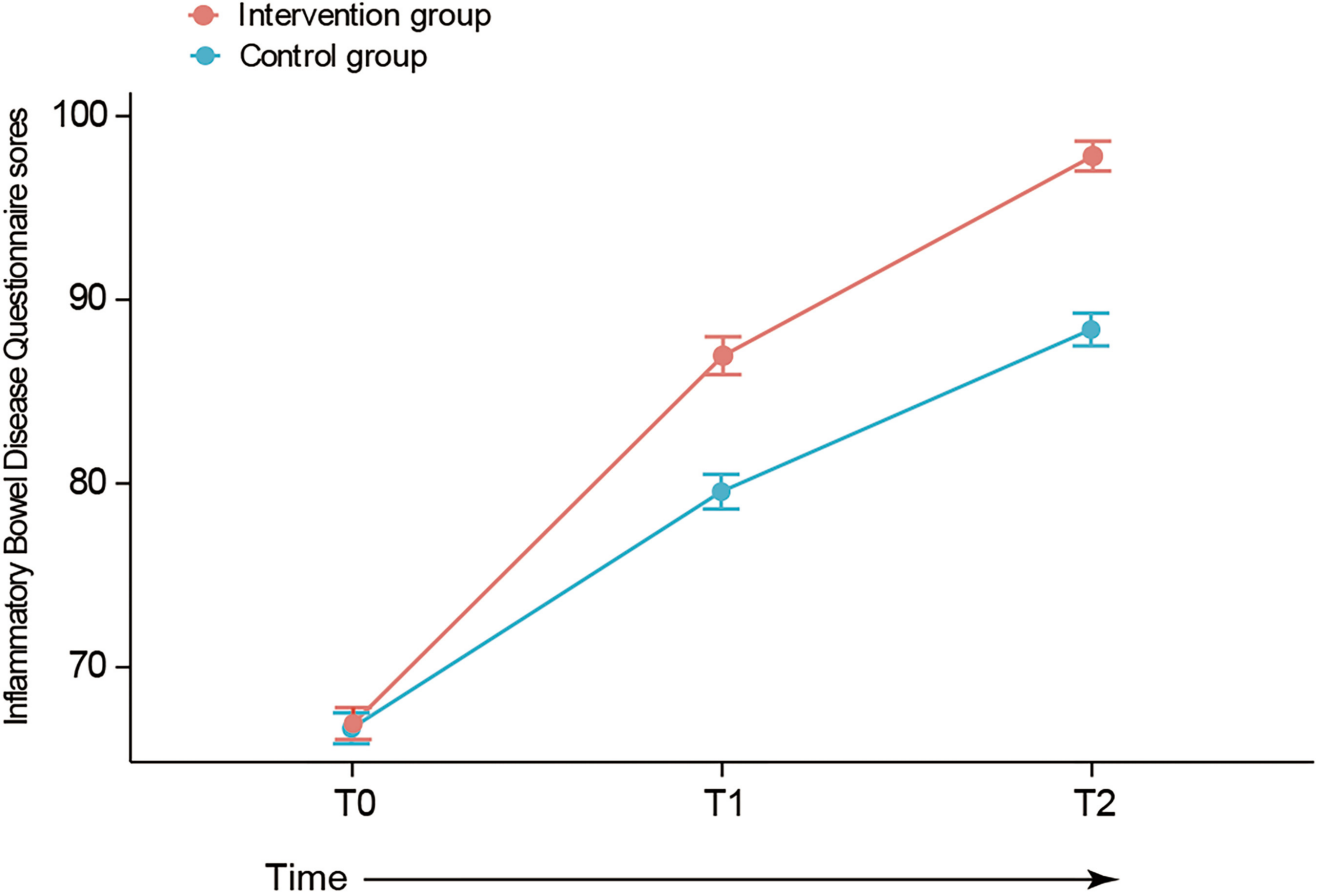
Trends in health-related quality of life. T0, 24 h of admission; T1, day of discharge; T2, 30 days post-discharge.

As illustrated in Figure 3, both groups demonstrated progressive improvements in IBDQ scores. From a comparable baseline (T0: intervention, 66.9 vs. control, 66.7), the intervention group achieved higher scores at discharge (T1: 87.0 vs. 79.6) and a greater sustained improvement at the 30-day follow-up (T2: 97.8 vs. 88.4).

Linear mixed-effects modeling confirmed a statistically significant group-by-time interaction (all *P* < 0.001). While a significant increase from baseline was observed in both groups at T1 (β = 12.9, *P* < 0.001) and T2 (β = 21.7, *P* < 0.001), the intervention group showed a significantly greater incremental benefit. The estimated additional effect of the intervention was 7.1 points (95% CI: 3.6–10.6) at T1 and 9.2 points (95% CI: 5.7–12.7) at T2 compared to the control group. Detailed results are presented in Table 4. Sensitivity analyses adjusting for baseline characteristics (age, gender, family annual income, marital status, educational level, disease duration, disease type, and reason for admission) produced virtually identical estimates (see Table 5), confirming the robustness of the findings.

**TABLE 4:** Results of the linear mixed-effects model for health-related quality of life.

Parameter	Comparison	Estimate (95% CI)	SE	*t*	*P*
Group	Intervention vs. Control	0.3 (-2.2, 2.7)	1.26	0.212	0.832
Time	T1 vs. T0	12.9 (10.4, 15.4)	1.26	10.239	< 0.001
	T2 vs. T0	21.7 (19.2, 24.2)	1.26	17.247	< 0.001
Group × Time	Intervention × T1	7.1 (3.6, 10.6)	1.78	4.007	< 0.001
	Intervention × T2	9.2 (5.7, 12.7)	1.78	5.155	< 0.001

Unadjusted for covariates.

**TABLE 5:** Results adjusted of the linear mixed-effects model for health-related quality of life.

Parameter	Comparison	Estimate (95% CI)	SE	*t*	*P*
Group	Intervention vs. Control	0.2 (-2.0, 2.4)	1.12	0.179	0.858
Time	T1 vs. T0	12.8 (10.5, 15.1)	1.17	10.940	< 0.001
	T2 vs. T0	21.6 (19.3, 23.9)	1.17	18.462	< 0.001
Group × Time	Intervention × T1	7.0 (3.7, 10.3)	1.68	4.167	< 0.001
	Intervention × T2	9.1 (5.8, 12.4)	1.68	5.417	< 0.001

Adjusted for age, gender, family annual income, marital status, educational level, disease duration, disease type, and reason for admission.

## 4 Discussion

This two-phase, mixed-methods study successfully developed and validated a structured discharge planning program based on the Team STEPPS framework for patients with IBD. The findings indicate that this program can significantly improve key preparedness metrics at discharge and confer sustained benefits on patients’ quality of life, with several aspects warranting further discussion.

The development of the Team STEPPS-based program was grounded in a rigorous modified Delphi process involving a 16-member multidisciplinary expert panel (23). Through two rounds of anonymous consultation, the panel systematically integrated current evidence to achieve a high level of consensus. All program components met stringent predefined criteria, including a mean importance score exceeding 4.0, a coefficient of variation below 0.25, and a high expert authority coefficient of 0.914, collectively affirming the robustness and credibility of the final protocol (24). The key methodological contribution was the operationalization of abstract Team STEPPS principles into a concrete, six-phase protocol specifically tailored for IBD discharge care. This consensus-driven intervention directly addresses transitional care needs through clearly defined team roles, structured communication tools, and phased tasks, thereby targeting the coordination failures inherent in conventional discharge planning. The embedded collaborative mechanisms are recognized factors for enhancing healthcare quality and patient safety (24). Consequently, the significant effects observed in the subsequent clinical trial validate this scientifically constructed and operationally feasible intervention scheme.

The significantly superior scores in discharge readiness and the quality of discharge teaching observed in the intervention group (*P* < 0.001) align with and extend previous evidence supporting structured, multidisciplinary approaches to transitional care (13, 25). While prior studies have established the importance of discharge planning, the present results specifically demonstrate that a standardized protocol, such as the Team STEPPS-based model, can effectively enhance both the perceived availability of support and personal coping ability, as well as the systematic delivery and content of patient education. We posit that the underlying mechanism for this improvement is the replacement of fragmented, often nurse-centric education with a coordinated, team-based strategy. This model ensures consistency, comprehensiveness, and a dual focus on the information provided (received content) and the effectiveness of its delivery, thereby addressing critical gaps frequently reported in conventional care (26, 27). The smaller, yet statistically significant, difference in the “needed content” subscale suggests that while the intervention better met perceived needs, fully aligning educational content with individual patient expectations remains a nuanced challenge, potentially requiring more personalized assessment approaches.

The progressive and significantly greater improvement in health-related quality of life (IBDQ scores) in the intervention group (*P* < 0.001), sustained at the 30-day follow-up, provides compelling evidence that optimizing discharge processes yields tangible, longer-term benefits for patient-reported outcomes. This finding corroborates the conceptual link between effective care transitions and sustained quality of life, a relationship suggested but less frequently quantified in chronic disease management (7, 28). The linear mixed-effects model confirmed that the intervention provided an independent, additive benefit beyond the natural recovery trajectory post-discharge. We speculate that this sustained effect is mediated through the intervention’s multifaceted nature: improved discharge readiness likely empowered patients for immediate self-management, while high-quality, reinforced teaching and structured follow-up created a foundation for sustained confidence and treatment adherence (29). Notably, the increasing absolute difference in scores from discharge to the 30-day follow-up implies that the intervention’s value may amplify over time, possibly by mitigating early post-discharge complications or anxiety that can undermine recovery (7, 30). This supports the perspective that investment in a robust discharge system is not merely a procedural endpoint but a critical determinant of the medium-term recovery trajectory.

This study has several limitations. As a single-center pilot with a relatively modest sample size, the generalizability of our findings requires confirmation in larger, multi-center trials. The 30-day follow-up period captured short-term effects on patient preparedness and early quality of life but was insufficient to assess the intervention’s impact on medium- to long-term clinical endpoints. Future studies should extend follow-up duration and incorporate health economic evaluations. Furthermore, the study primarily relied on patient-reported outcomes; integrating objective clinical indicators, such as biomarkers of disease activity, would provide a more comprehensive assessment. While the Team STEPPS-based intervention was successfully implemented, we did not conduct a formal process evaluation to quantify the frequency and quality of teamwork tool usage, which should be incorporated in future implementation research. Finally, the sample size was also insufficient for meaningful subgroup analyses. Future multicenter trials should examine whether the intervention’s effectiveness varies by admission diagnosis and whether further tailoring is warranted for specific clinical scenarios. Despite these limitations, this study represents a critical step toward developing an evidence-based transitional care model for IBD. The positive results establish a solid foundation for subsequent program optimization and broader clinical dissemination.

## 5 Conclusion

This two-phase study successfully developed, through expert consensus, and preliminarily validated a Team STEPPS-based discharge planning program for IBD patients. The intervention significantly enhanced key transitional care outcomes, including discharge readiness, discharge teaching quality, and health-related quality of life, supporting its potential value in improving post-hospitalization care for this population. These promising results warrant confirmation in larger, multi-center trials with extended follow-up periods and embedded process evaluations.

## Data Availability

The raw data supporting the conclusions of this article will be made available by the authors, without undue reservation.
